# Poverty Does Make Us Sick

**DOI:** 10.5334/aogh.2357

**Published:** 2019-03-13

**Authors:** Nazim Habibov, Alena Auchynnikava, Rong Luo

**Affiliations:** 1 University of Windsor, CA

## Abstract

This study evaluates the direct causal effects of household wealth on health. We discuss several specific mechanisms that that could relate poverty with worse health and hypothesize that poverty will undermine population health. This hypothesis was tested based on data drawn from a recent cross-country survey in 12 post-Soviet countries and Mongolia using classic regression (OLS) and instrumental variable 2SLS regressions. The results indicate that poverty does indeed lead to worsening health. This negative effect of poverty on health remains unchanged after controlling for a wide range of individual characteristics, healthcare performance indicators, trust in individuals, government, parliament, and political parties, as well as country-level unobserved characteristics. Using an instrumental variable increases our confidence in being able to isolate the effects of poverty on health status and confirms that our results are not due to endogeneity. In addition, the strong negative effect of poverty on health remains robust to the use of a set of country-level aggregated indicators (e.g. GDP and Gini) instead of country dummies, the employment of a subjective self-assessment indicator of poverty instead of an objective one, and an alternative conceptualization of health status as a binomial variable (for bad and very bad health) instead of a continuous one.

## Introduction

At the center of the debate about the social determinants of health has been the issue of the effect of wealth on the health status of individuals. Of specific concern has been the question of the extent to which individuals from poorer households are less healthy than individuals from wealthier households [1,2,3]. However, findings within the research literature about the links between wealth and health status have been found to be far from consistent. Grossman (1972) [4] suggested early on that income has a positive effect on health since increases in wages are associated with improvements in health status, and more recent studies have concurred by reporting that individuals who reside in poorer households tend to be less healthy due to lack of resources and psychosocial stress [5,6,7,8]. In contrast, reverse causality is also a plausible explanation insofar as healthier individuals are able to work more productively and may therefore lift their households out of poverty, while negative health shocks, for instance, acute and chronic illness, may plunge households into poverty [9,10,11,12]. Additionally, causality may affect income and health due to unobserved confounders, for instance, latent individual personality traits, individual time preferences, or stages of epidemiological transition [13,14,15]. Likewise, it is also conceivable that the relationship between poverty and health is the result of problems in measurement error, especially when poverty or health status are measured by subjective variables [16,17]. A final possibility is that although there may be a relationship between poverty and health, it may either not be statistically significant or not significant enough in magnitude to affect policy [18,19,20].

With these options in mind, the purpose of this study is to assess the effects of household wealth on health in post-Soviet countries. These countries represent a particularly interesting case for studying the effects of household wealth on health status for three main reasons. First, the collapse of communism led to a political, social, and economic crisis that was more profound and prolonged in the countries of the former Soviet Union than in other countries of the former Eastern block and was accompanied by sharp increases in poverty and income inequality [21,22,23]. Even now, more than 15 years after transition commenced, the countries of the former Soviet Union are significantly lagging behind other countries of the former Eastern block in terms of their levels of socio-economic development and the speed of their reforms [24,25,26]. Second, the health status of their populations have seriously deteriorated inasmuch as the countries of the former Soviet Union experienced stagnation and even decline in life expectancy, higher rates of infant and child mortality, tuberculosis, alcoholism, drug abuse, STD, and HIV/AIDS [27,28,29,30,31]. Third, the economic crisis has led to a chronic underfunding of public health care, which in turn has lead to sharp increases in the amount and frequency of official out-of-pocket expenditures and informal under-the-counter payments [32,33,34]. Underfunding has also led to a shortage of modern equipment, lack of training in contemporary procedures and technologies, as well as to sharp reductions in satisfaction with public healthcare [35,36,37].

Resulting from the above-discussed circumstances, poverty worsens health in post-Soviet countries in seven main ways. First, individuals residing in lower income households are less likely, when they are sick or when it is required by protocol such as during the pregnancy, to use healthcare than are individual from higher income households [38,39,40,41]. Second, individuals residing in lower income households have limited access to more advanced, up-to-date, and specialized health procedures and services, and this forces them to use less advanced procedures and services [42,43,44,66]. Third, poorer households often cannot afford to buy prescribed medications [45,46]. Fourth, the poor have no choice but to seek consultations with less specialized healthcare personnel. For instance, poor patients are more likely to have to consult nurses instead of doctors [47,48]. Fifth, patients from less affluent households are more likely have to pay informal under-the-counter payments in healthcare settings [36]. Sixth, poverty is associated with considerable stress, depression, and thus overall strong negative psychological effects [49,50]. Finally, poverty is associated with hazardous home environments and limited opportunities to maintain a healthy lifestyle [51,52]. Thus, the discussion above allows us to articulate the following testable hypothesis:

*Hypothesis 1:* Individuals from poorer households will have worse health. Keeping this in mind, let us now turn to the methods section of our study.

## Methods

### Data

We use data from the 2016 Life-in-Transition survey (LITS) conducted by the European Bank for International Development [24,25]. LITS is a comprehensive cross-sectional socio-economic population survey that collects data using a multistage clustering design. Approximately 1500 respondents were selected for face-to-face interviews with trained interviewers. A detailed description of the LITS, which includes a discussion of the sample design, the participation rates from each country, response rates, and the socio-demographic characteristics of the sample, is provided by Habibov and Cheung (2017) [34] and Habibov, Cheung, and Auchynnikava (2017) [40]. Since the LITS allow for full cross-country comparability, the survey has been used in a variety of comparative health research (e.g. Bauer et al., 2017; Nikolova and Sanfey, 2016) [53,54]. Here, we focus on Armenia, Azerbaijan, Belarus, Georgia, Kazakhstan, Kyrgyzstan, Moldova, Russia, Tajikistan, Ukraine, and Uzbekistan. Mongolia, which was not formally part of the former Soviet Union, is also included due to the significant similarities with other countries in our analysis.

### Outcome, predictor, and covariates

Our outcome of interest is health status. The LITS asked respondents to report their health status according to an ordinal scale that ranged from 1 = “very good” to 5 = “very bad”. We take the lead from the previous literature in interpreting the answers to the subjective health questions as a summary of true health status, which is assumed to be a continuous variable that is determined by the main predictor of interest and its covariates [6,55].

Our predictor of interest is poverty. In line with past research, we focus on expenditure rather than income as a measure of poverty for two main reasons [43]: (a) income, as compared with expenditures, is severely underreported in surveys conducted in post-communist countries; (b) income often fluctuates sharply due to wage and benefit arrears, and people may continue to use their savings to compensate for this fluctuation. Also, in alignment with previous studies, we focus on household expenditures since households pool their resources when needed, for example, when expensive prescribed medication or specialized health procedures are to be paid for a household member [16]. Thus, we compute total household expenditures for each household. To adjust expenditures by household size, the total expenditure of a household is divided by the square root of the number of people in the household [43]. Finally, to be able to directly compare households across countries, we divide them into 5 equal quintiles, where quintile 1 represents the wealthiest 20% of households in every country and quintile 5 represents the poorest 20% [34].

Given the nature of our predictor and outcome variables, we expect that moving from the wealthiest quintile 1 towards the poorest quintile 5 will be led to worsening health status from 1 = excellent health to 5 = very bad health.

We control for several blocks of covariates. First, we control for the influence of respondent characteristics such as age, gender, education, marital and employment status, and living in rural areas [55]. Second, we control for public healthcare performance as experienced by LITS respondents: (1) the frequent and unjustified absence of doctors, (2) disrespectful treatment by staff, (3) no necessary medication available, (4) long waiting time, (5) facilities are not clean, and (6) informal payments required for services that should be free. Third, we control for the effects of trust: (a) generalized trust in other people, (b) trust in government; (c) trust in parliament, and (d) trust in political parties [40]. Given that the influence of healthcare performance and trust can be important at the community level, we average the influence of healthcare performance and trust at the community level [55].

Lastly, to control for country-level unobserved characteristics such as differences in healthcare design and effectiveness, and cross-cultural variations in health status, we use country dummies as covariates. The main advantage of using country dummies is that their intercept will fully absorb the country-level unobserved characteristics such as variation in understanding health status, expectations regarding the performance of the healthcare system, and political traditions [56]. Consequently, country dummies are best positioned to capture the country-level influence on health status [40]. The disadvantage of using country dummies is that they are correlated with country-level variables (e.g. GDP) and hence cannot be used in the same regression model [40,56]. We address this disadvantage in the robustness analysis section below.

The descriptive statistics for sample could be found in Electronic Supplementary Appendix A.

### Model set-up

A straightforward approach to estimating the effects of household wealth on an individual’s health status is to estimate a classic OLS regression where health status is the outcome, and household wealth is one of the correlates. However, such a naive approach will lead to biased results due to problems of reverse causality, unobserved confounders, and measurement error. Consequently, the true effect of the predictor on the outcome could either be underestimated or overestimated, but the direction of such bias would be theoretically ambiguous and could not be known a priori [57]. The most effective way to address the problems of reverse causality, unobserved confounders, and measurement error is to estimate an instrumental variable 2SLS regression model [58,59,60]. Formally, the 2SLS consists of two OLS regression equations. In the first-stage equation, household wealth is regressed on the covariates and the instruments. In the second, often called a main-stage equation, health status is regressed on the covariates and on the value of household wealth, which was estimated in the first-stage equation.

Good instruments should simultaneously satisfy three conditions. First, they should be relevant, meaning that they should be strongly correlated with the predictor. Second, they should be valid, meaning that they should not be directly correlated with the outcome. Third, the instrumental variable equation should pass the test for endogeneity, which would reveal the presence of endogeneity in the classic OLS model and hence would reject the results of the classic model in favour of the 2SLS results.

The existing literature indicates that owning your dwelling (versus being a tenant who either pays or does not pay rent) and having access to the internet at your dwelling could be good instruments since they are expected to be associated with being from a wealthier household, while there is no compelling reason to imagine that these instruments would directly affect the self-ranked health status of the individual [61,62].

As suggested by the literature, we test whether our instruments satisfied all three of the above-discussed conditions [57]. We commence by testing for instrument relevance, which stipulates that the instruments should be correlated with the predictor. We begin by using first-stage F-statistics that are statistically significant and considerably larger than the rule of thumb value of 10 in all our estimations. This signals that the correlation between the instruments and the predictor is not weak. Second, our F-statistics are considerably larger than the Stock and Yogo’s critical values. Third, the results of first-stage regression reported in Electronic Supplementary Appendix B show that every instrument is strongly correlated with the outcome in the expected direction. We continue by testing for instrument validity, which stipulates that the instruments should not have any significant influence on the outcome variable other than through the predictor. To test for instrument validity, we use the Sargan and Basman tests. The results of both tests are not statistically significant in all estimated models, indicating that the instruments do not jointly exert a direct influence on the outcome variable. In the end, to formally establish the presence of endogeneity, we use the Durbin and Wu-Hausman tests for endogeneity. The statistically significant results of both tests in all estimated models signal that the classic OLS model is inconsistent because of endogeneity, and as such, justifies the use of the instrumental variable approach.

### Analytic strategy

The explanations of variation in health status used in this study may be correlated with each other. In order to isolate the influence of potentially correlated variables, our research strategy consists of the estimation of several consecutive 2SLS models. Our first model includes regressing health status on household wealth while controlling for individual and household characteristics only. This is our baseline model and serves as a benchmark for all the following models. The second, third, and fourth models expand upon this first baseline model by consecutively incorporating (a) public healthcare performance, (b) individual and institutional trust, and (c) public healthcare performance and trust at the community level. Thus, the fourth model is our main model and incorporates all the covariates. In addition, we estimate an OLS model with the same set of variables as our main model to compare the significance, direction, and magnitude of the effect between our main 2SLS model and the OLS model.

## Results

### Main results

The results of the baseline model are shown in the first column of Table 1. The results suggest that lower household expenditures lead to a significant reduction in health status (*β* = 0.218; *p-value* = 0.000). Among the covariates, being older, a woman, and unemployed is associated with worsening health. In contrast, having a university education and residing in an urban area is associated with improving health.

**Table 1:** Main results (outcome variable from 5 = very bad health to 1 = excellent health).

Variables	Model 1	Model 2	Model 3	Model 4	Model 5

	2SLS	2SLS	2SLS	2SLS	OLS

Household wealth (1 = the wealthiest quintile and 5 = the poorest quintile)	0.218***	0.258***	0.263***	0.259***	0.020**
	(0.025)	(0.035)	(0.036)	(0.036)	(0.007)
Age	0.018***	0.018***	0.018***	0.018***	0.019***
	(0.001)	(0.001)	(0.001)	(0.001)	(0.001)
Female	0.113***	0.078***	0.081***	0.081***	0.086***
	(0.015)	(0.021)	(0.022)	(0.022)	(0.020)
University education	–0.043*	–0.008	–0.018	–0.017	–0.113***
	(0.019)	(0.027)	(0.028)	(0.028)	(0.022)
Married	0.004	–0.043	–0.041	–0.042	–0.093***
	(0.017)	(0.023)	(0.025)	(0.025)	(0.022)
Unemployment	0.195***	0.204***	0.207***	0.209***	0.291***
	(0.019)	(0.026)	(0.028)	(0.028)	(0.023)
Urban area	–0.088***	–0.101***	–0.108***	–0.103***	–0.001
	(0.019)	(0.027)	(0.028)	(0.028)	(0.022)
Frequent unjustifiable absence of healthcare personnel		–0.017	–0.007	0.020	–0.007
		(0.034)	(0.036)	(0.042)	(0.039)
Disrespectful treatment by healthcare personnel		0.062*	0.036	0.035	0.052
		(0.030)	(0.032)	(0.036)	(0.033)
No required drug available		0.110***	0.106***	0.110***	0.124***
		(0.026)	(0.027)	(0.032)	(0.029)
Healthcare facilities not clean		–0.007	–0.012	0.030	0.025
		(0.042)	(0.044)	(0.053)	(0.048)
Payments required for services that should be free-of-charge		0.183***	0.181***	0.151***	0.135***
		(0.028)	(0.030)	(0.034)	(0.032)
Long waiting time		0.016	0.008	0.054	0.045
		(0.024)	(0.026)	(0.030)	(0.028)
Generalized trust in other people			–0.049*	–0.053*	–0.062*
		(0.024)	(0.026)	(0.024)
Trust in government			–0.037*	–0.033*	–0.032*
		(0.015)	(0.016)	(0.015)
Trust in parliament			–0.039*	–0.030	–0.027
		(0.017)	(0.017)	(0.016)
Trust in political parties			–0.006	–0.006	–0.002
			(0.013)	(0.014)	(0.013)
Community generalized trust				0.042	–0.038
				(0.060)	(0.054)
Community trust in government				–0.016	–0.032
				(0.052)	(0.048)
Community trust in parliament				–0.064	–0.033
				(0.060)	(0.055)
Community trust in political parties				0.021	0.028
				(0.035)	(0.032)
Community level frequency of unjustified absence of doctors				–0.046	0.021
				(0.086)	(0.079)
Community level disrespectful treatment by healthcare personnel				0.003	–0.033
				(0.085)	(0.078)
				–0.004	0.021
Community level no required drug available				(0.062)	(0.058)
				–0.139	–0.106
Community level healthcare facilities not clean				(0.100)	(0.092)
				0.103	0.051
Community level free services that are charged for				(0.067)	(0.061)
				–0.157**	–0.179***
Community level long waiting time				(0.059)	(0.054)
					
Country dummies included	YES	YES	YES	YES	YES
Number of cases	13233	6605	5851	5851	5851
Wald test of equality of all regression coefficients in the main stage *χ*^2^	4513.26***	2539.60***	2331.99***	2369.15***	
F-statistic of equality of all regression coefficients					71.86***
*Testing instrument relevancy*					
First-stage robust F statistic	299.04***	158.40***	146.50***	147.59***	
Minimum eigenvalue statistic	299.04	158.40	146.50	147.59	
Stock and Yogo’s statistic	19.93	19.93	19.93	19.93	
*Testing instrument validity*					
Sargan *χ*^2^	0.01	0.02	0.07	0.16	
Basmann *χ*^2^	0.01	0.02	0.07	0.16	
Testing engdogeneity					
Durbin *χ*^2^	80.59***	61.09***	55.85***	54.34***	
Wu-Hausman F statistic	80.96***	61.41***	56.09***	54.47***	

*Notes:* Data rounded up. Regression coefficients and robust standard errors are reported for 2SLS and OLS. Significance level: * p < 0.05; ** p < 0.01; *** p < 0.001. Country-dummies are not shown to conserve space.

Healthcare performance characteristics are added in Model 2. After incorporating these characteristics, we note that lower household expenditures still lead to worsening health (*β* = 0.258; *p-value* = 0.000). Among healthcare performance characteristics, being treated disrespectfully by healthcare personnel, not having access to required drugs, and being asked to pay unofficial under-the-counter out-of-pocket fees for services that should be provided free-of-charge, are correlated with poorer health.

Trust characteristics are added in Model 3. Incorporating these characteristics does not change the direction and significance of the impact of the reduction in household expenditures on worsening health (*β* = 0.263; *p-value* = 0.000). Besides, trust in government and parliament is correlated with better health. Finally, healthcare performance and trust at the community level are added in Model 4. Their inclusion does not change the direction and significance of the impact of a reduction in household wealth on worsening health (*β* = 0.259; *p-value* = 0.000). As well, experiencing long waiting times for access to healthcare in the community is correlated with worsening health. At the same time, the effect of education, and trust in government and parliament both lost significance.

We also conducted a Wald test of equality on the regression coefficients. The null hypothesis of the Wald test is that all regression coefficients in a given model are equal to each other. Significant results of these tests lead to a rejection of the notion of equality of the coefficients and suggest that the effect of every coefficient in the model is significantly different from the effects of other coefficients in the model.

Finally, it is instructive to evaluate the effects of poverty on health if we do not address endogeneity through the 2SLS. Therefore, we estimate the OLS with exactly the same set of covariates as in our main Model 4. The results of OLS are reported in Model 5. They suggest that poverty will still have a significant detrimental effect on health (*β* = 0.020; *p-value* = 0.000). However, failure to address endogeneity by OLS leads to a significant, almost tenfold, underestimation of the true effect of poverty on health. The magnitude of such an underestimation is in line with that which was reported in other studies, and is likely due to endogeneity [5,58,59].

### Robustness of the main results

We test the robustness of our results for (a) a different conceptualisation of the predictor, household wealth; (b) a different set of covariates; (c) a different conceptualization of the outcome, health status. The purpose of the robustness analysis is to assess whether our main results will change in term of the significance and direction of the effect as a result of using a different conceptualisation of the predictor, a different set of covariates, and a different conceptualization of the outcome.

We commence by testing for a different conceptualisation of the predictor. Instead of using quintiles of household wealth, we use the individual’s own subjective assessment of poverty. The LITS asks respondents the following questions about their subjective assessment of their household’s position on the relative welfare ladder: “Please imagine a ten-step ladder where on the first step stand the wealthiest 10% of people in our country, and on the tenth stand the poorest 10% people in our country. On which step of the ten is your household today?” Thus, we re-estimate our main Model 4 using the answer to this question as the predictor. The results are reported in Model 6 of Table 2 and suggest that lower subjective wealth also leads to worsening health (*β* = 0.317; *p-value* = 0.000).

**Table 2:** Robustness analysis of main results.

Variables	Model 6	Model 7	Model 8

	2SLS	2SLS	ivprobit

Subjective assessment of household wealth (1 = the wealthiest 10% of households in the country and 10 = the poorest 10% of households in the country)	0.317***		
	(0.047)		
Household wealth (1 = wealthiest quintile and 5 = poorest)		0.280***	0.085***
		(0.038)	(0.013)
GDP per capita		0.000***	
		(0.000)	
GDP growth rate		–0.008	
		(0.007)	
Gini		0.013***	
		(0.003)	
Current health expenditure		0.042***	
		(0.010)	
Out-of-pocket expenditure		0.003*	
	(0.001)	
Age	0.016***	0.018***	0.004***
	(0.001)	(0.001)	(0.000)
Female	0.092***	0.072**	–0.002
	(0.024)	(0.022)	(0.009)
University education	–0.012	–0.015	–0.003
	(0.030)	(0.028)	(0.012)
Married	–0.005	–0.021	–0.023*
	(0.029)	(0.025)	(0.010)
Unemployed	0.206***	0.203***	0.083***
	(0.030)	(0.029)	(0.012)
Urban area	–0.024	–0.135***	–0.019
	(0.025)	(0.029)	(0.011)
Frequent unjustifiable absence of healthcare personnel	–0.002	0.023	0.004
	(0.045)	(0.043)	(0.018)
Disrespectful treatment by healthcare personnel	0.027	0.033	0.024
	(0.038)	(0.037)	(0.015)
No required drug available	0.077*	0.110***	0.044***
	(0.035)	(0.033)	(0.013)
Healthcare facilities not clean	0.022	0.032	0.019
	(0.056)	(0.054)	(0.022)
Payments required for services which should be free-of-charge	0.107**	0.157***	0.036*
	(0.037)	(0.035)	(0.014)
Long waiting time	0.033	0.056	0.003
	(0.032)	(0.031)	(0.013)
Generalized trust in other people	–0.014	–0.050	0.010
	(0.029)	(0.027)	(0.011)
Trust in government	–0.005	–0.034*	–0.015*
	(0.018)	(0.017)	(0.007)
Trust in parliament	–0.022	–0.028	–0.007
	(0.018)	(0.018)	(0.007)
Trust in political parties	0.013	–0.006	–0.004
	(0.015)	(0.015)	(0.006)
Community generalized trust	–0.012	0.027	0.023
	(0.063)	(0.060)	(0.026)
Community trust in government	–0.077	–0.060	0.019
	(0.056)	(0.050)	(0.022)
Community trust in parliament	0.031	0.036	–0.030
	(0.064)	(0.060)	(0.026)
Community trust in political parties	0.019	–0.007	0.002
	(0.037)	(0.035)	(0.015)
Community-level frequency of unjustified absence of doctors	0.062	–0.082	–0.029
	(0.091)	(0.087)	(0.037)
Community level disrespectful treatment by healthcare personnel	–0.058	–0.011	0.002
	(0.090)	(0.086)	(0.037)
Community level no required drug available	–0.048	–0.048	0.001
	(0.067)	(0.063)	(0.027)
Community level healthcare facilities not clean	0.086	–0.086	–0.065
	(0.111)	(0.101)	(0.043)
Community level free services that are charged for	0.123	0.017	0.049
	(0.071)	(0.065)	(0.029)
Community level long time waiting	–0.170**	–0.165**	–0.060*
	(0.062)	(0.059)	(0.026)
Country dummies included	YES	NO	YES
Number of cases	5791	5851	5862
Wald test of equality of all marginal effects in the main stage *χ*^2^	2072.25***	2218.50***	1501.40***
*Testing instrument relevancy*			
First-stage robust F statistic	63.41***	138.32***	146.09***
Minimum eigenvalue statistic	63.41	138.32	146.09
Stock and Yogo’s statistic	19.93	19.93	19.93
*Testing of instrument validity*			
Sargan *χ*^2^	0.75	0.01	2.46
Basmann *χ*^2^	0.75	0.01	2.45
*Testing engdogeneity*			
Durbin *χ*^2^	44.29***	61.17***	
Wu-Hausman F statistic	44.32***	61.42***	
Wald test of equality of all marginal effects in the main stage *χ*^2^			33.82***

*Notes:* Data rounded up. Outcome in Model 6 and 7 is ordinal from 5 if the individual reported very bad health to 1 if excellent health. Outcome variable in Model 8 is binomial and equal to 1 if the individual reported bad or very bad health. Regression coefficients and robust standard errors are reported for 2SLS, while marginal effects and robust standard errors are reported for ivprobit. Significance level: * p < 0.05; ** p < 0.01; *** p < 0.001. Country-dummies are not shown to conserve space.

The next step is to test the robustness of our results for different sets of covariates. Here, we drop country dummies and instead use country-level aggregated characteristics, namely, Gross Domestic Product per capita, percentage of annual Gross Domestic Product growth, Gini coefficient, current health expenditure as a percentage of Gross Domestic Product, and percentage of out-of-pocket payments in total current health expenditures. The results are reported in Model 7 of Table 2 and suggest that using country-level aggregated characteristics instead of country dummies will not considerably alter the results as compared with our main model (*β* = 0.280; *p-value* = 0.000 vs. *β* = 0.259; *p-value* = 0.000).

Finally, we follow an alternative tradition is studying health status and reconceptualise health status as a binary variable (1 = bad and very bad health). Since the outcome variable is binary, the appropriate instrumental variable model is ivprobit. The first-stage of ivprobit is OLS where quintiles of household wealth are regressed on covariates and instruments. The second, often called main-stage equation, is binomial probit where binomial health status is regressed on the covariates and on the value of household wealth, which is estimated in the first-stage by OLS. The results of ivprobit estimation are reported in Model 8 of Table 2. To facilitate the discussion regarding the results of non-linear ivprobit, all coefficients are transformed to marginal effects. As shown, residing in a household with lower wealth increased the likelihood of reporting bad or vary bad health by approximately 9 percentage points (*α* = 0.085, *p-value* = 0.000). Overall, the results of robustness analysis allow us to conclude that in term of the significance and direction of the effect, our main results are robust to different conceptualisations of poverty and health status, and to different sets of covariates.

## Limitations

Our analysis is not without its limitations. First, the small country sample prevents us from conducting a country-by-country analysis. Second, although there is no reason to believe that our instruments have a direct effect on the outcome, the possibility of such an effect remains a potential pitfall for any IV analysis. Third, the self-rated health measure was not validated across countries and may be different across the countries under investigation. Nevertheless, the possible across-country bias was reduced by our inclusion of country dummies and country-level indicators of socio-economic development.

## Conclusion

Given the inconsistency of the results of previous studies, interpretations regarding the relationship between poverty and health have been the subject of much controversy. With this in mind, the current study has focused on evaluating the direct causal effects of poverty on health status in the countries of the former Soviet Union and Mongolia. In the introductory part of our paper, we discussed several specific mechanisms that could translate poverty into worse health within the context of post-Soviet countries, and we hypothesized that poverty will undermine population health. We then tested this hypothesis based on data drawn from a recent comparable survey using classic and instrumental variable regressions.

We found that poverty does indeed lead to worsening health. The negative effects of poverty on health remained unchanged even after controlling for a wide range of individual characteristics, public healthcare performance indicators, trust of individuals, government, parliament, and political parties, as well as country-level unobserved characteristics. Using instrumental variable regression increased our confidence that we were able to isolate the effects of poverty on health status and that our results are not a phenomenon of endogeneity. In addition, the strong negative effects of poverty on health remain robust to using a set of country-level aggregated indicators (e.g. Gross Domestic Product and Gini) instead of country dummies, the subjective self-assessed indicator of poverty instead of the objective one, and the alternative conceptualization of health status as a binomial variable (for bad and very bad health) instead of the continuous one.

Our findings suggest that wealthier people tend to be healthier since an increase in household expenditures leads to improved health. As well, our findings also suggest that increases in poverty will have detrimental effects on the health of individuals. As such, worsening health will reduce their productive capacity, limit their participation in the labour market, and will force them to pay more for healthcare and curb investments in their children’s health, thus creating a vicious circle. Consequently, the main policy implication of our findings is that the implementation of policies whose intent is to ameliorate population health will be well supported by policies aimed at reducing poverty.

Our findings also suggest that classic models (e.g. ordinary least square) systematically underestimate the true effects of poverty on health. Such a significant underestimation of the true effect of poverty on health does not just represent a statistical nuance. The underestimation of the detrimental effects of poverty on health undermines the interests of policy makers, healthcare administrators, and international donors with respect to the role of poverty in determining population health.

The fact that endogeneity leads to a considerable underestimation of the true effects of poverty on health has three main implications. On the one hand, reverse causality appears not to be a main problem in our estimation of a poverty-health link because significant reverse causality would more likely lead to an overestimation of the true effects of poverty on health through classic regression models [57]. On the other hand, the underestimation of the impact of poverty on health could be caused by measurement error in gauging subjective health status if such a measurement error is systematically associated with being poor [63]. As Sen has explained, “A population …which has widespread health problems as a standard condition of existence, can have very low perception of being medically ill” [64 p18]. In line with this reasoning, if poorer people perceive their health to be better than it really is due to a form of psychological adaptation to their relative ill-health, then classic regression methods such as OLS will underestimate the true effects of poverty on health [16]. In addition, another possible explanation for the fact that the true effect of poverty on health is underestimated by classic regression is the significant effect of unobserved confounders, for example, latent individual personality traits or individual time preferences [13,14,15].

Finally, the influence of covariates within this study is in line with those found in previous studies. Thus, being older, a woman, and unemployed are all associated with worsening health–these results are in line with those reported in previous studies that were also conducted in post-soviet countries [65]. Lack of required medication, informal under-the-counter out-of-pocket payments, and longer waiting periods for healthcare appointments in the community are also associated with worsening health [36,45,46]

## Additional Files

The additional files for this article can be found as follows:

10.5334/aogh.2357.s1 Supplementary 1. Appendix A.

10.5334/aogh.2357.s2 Supplementary 2. Appendix B.
