# Supplementary material for: Poverty Does Make Us Sick
Source: Ann Glob Health. 2019 Mar 13;85(1):33. doi: 10.5334/aogh.2357 (PMC6634464; doi:10.5334/aogh.2357)
Supplement: Supplementary 1. — Appendix A. [file agh-85-1-2357-s1.pdf]

Appendix A

Table A1. Descriptive statistics

Variable	Definition	Mean	Std. Dev.	Min	Max	Source of data
Outcome variable						
Health status	Ordinal variable from 1 to 5, where 1= excellent health to 5= very bad health	2.679	0.884	1	5	LITS
Predictor						
Household wealth	Ordinal variable from 1 to 5, where 1 = the wealthiest 20% of households in the country and 5 is the poorest 20% of the households in the country	3.006	1.419	1	5	LITS
Household characteristics						
Age	Continuous variable of Age of the primary respondent	45.461	16.390	18	95	LITS
Female	Binary variable = 1 if a respondent reports is women	0.589		0	1	LITS
University education	Binary variable = 1 if a respondent has university degree or above	0.256		0	1	LITS
Married	Binary variable = 1 if a respondent is married	0.653		0	1	LITS
Unemployment	Binary variable = 1 if a respondent unemployed	0.360		0	1	LITS
Urban area	Binary variable = 1 if a respondent lives in urban area in a given country	0.546		0	1	LITS
Public healthcare performance						
Frequent unjustifiable absence of healthcare personnel	Binary variable = 1 if a respondent reported frequent and unjustified absence of doctors for the last 12 months	0.138		0	1	LITS
Disrespectful treatment by healthcare personnel	Binary variable = 1 if a respondent reported being treated disrespectfully by healthcare personnel	0.180		0	1	LITS
No required drug available	Binary variable = 1 if a respondent reported no required drugs was available in public healthcare facility	0.271		0	1	LITS
Healthcare facilities not clean	Binary variable = 1 if a respondent reported that healthcare facilities not clean	0.080		0	1	LITS
Payments required for services which should be free-of-charge	Binary variable = 1 if a respondent reported that payments required for services that should be free	0.215		0	1	LITS
Long waiting time	Binary variable = 1 if a respondent reported long waiting times	0.406		0	1	LITS

Attitudinal characteristics						
Generalized trust to other people	Continuous variable from 1 to 5, where 1= complete distrust people and 5 = complete trust people	0.301		0	1	LITS
Trust into government	Continuous variable from 1 to 5, where 1= complete distrust government and 5 = complete trust government	2.928	1.442	1	5	LITS
Trust into parliament	Continuous variable from 1 to 5, where 1= complete distrust parliament and 5 = complete trust parliament	2.812	1.416	1	5	LITS
Trust into political parties	Continues variable from 1 to 5, where 1= complete distrust political parties and 5 = complete trust political parties	2.618	1.367	1	5	LITS
Community characteristics						
Community generalized trust	Continues variable indicating generalized trust averaged at community level	0.301		0	1	LITS
Community trust into government	Continues variable indicating trust into government averaged at community level	2.941	1.084	1	5	LITS
Community trust into parliament	Continuous variable indicating trust into parliament averaged at community level	2.840	1.055	1	5	LITS
Community trust into political parties	Continuous variable indicating trust into political parties averaged at community level	2.658	0.963	1	5	LITS
Community level frequency of unjustified absence of doctors	Continues variable indicating frequency of unjustified absence of doctors averaged at community level	0.153		0	1	LITS
Community level disrespectful treatment by healthcare personnel	Continuous variable indicating being treated disrespectfully by healthcare personnel averaged at community level	0.187		0	1	LITS
Community level no required drug available	Continuous variable indicating no required drugs was available averaged at community level	0.270		0	1	LITS
Community level healthcare facilities not clean	Continuous variable indicating that healthcare facilities not clean averaged at community level	0.093		0	1	LITS
Community level free service that charge for payment	Continues variable indicating payments required for services that should be free averaged at community level	0.224		0	1	LITS
Community level long waiting time	Continuous variable indicating for long waiting times averaged at community level	0.393		0	1	LITS
Instruments						
Household owns dwelling	Binary variable = 1 if a respondent's household owns dwelling	0.913		0	1	LITS
Household has Internet access	Binary variable = 1 if a respondent's household has Internet access (inclusively on a smartphone)	0.542		0	1	LITS

Variables used in robustness analysis						
Subjective assessment of household wealth	Continuous variable from 1 to 10, where 1 = the wealthiest 10% of households in the country and 10 = the poorest 10% of households in the country	6.715	1.712	1	10	LITS
Poor health	Binary variable = 1 if a respondents reported bad and very bad health	0.138		0	1	LITS
GDP per capita	GDP per capita adjusted by purchasing power parity (PPP)	18615	9518	2985	35140	World Development Indicators by World bank[1]
GDP growth rate	Annual GDP growth rate (%)	2.329	2.300	-4.200	5.900	World Development Indicators by World bank[1]
Gini	Gini coefficient in percentage. Higher percentage indicates more inequality	31.400	4.59	23.700	42.800	World Inequality Database by WID[3]
Current health expenditure	Current health expenditure expressed as a percentage of GDP	7.006	1.592	3.900	10.200	Transmonee Database by Unicef[2]
Out-of-pocket expenditure	Share of out-of-pocket payments in total current health expenditures (%)	39.067	17.605	12.500	81.600	Transmonee Database by Unicef[2]

Note: Data are rounded up.

References

1. The World Bank. Word Development Indicators (n.d.). Retreived from https://datacatalog.worldbank.org/dataset/world-development-indicators.

2. Unicef. TransMonEE Database. (n.d.). Retrieved from http://transmonee.org/.

3. WID. World Inequality Database. (n.d.). Retrieved from http://wid.world
