# Supplementary material for: Poverty Does Make Us Sick
Source: Ann Glob Health. 2019 Mar 13;85(1):33. doi: 10.5334/aogh.2357 (PMC6634464; doi:10.5334/aogh.2357)
Supplement: Supplementary 2. — Appendix B. [file agh-85-1-2357-s2.pdf]

Appendix B. First-stage for results reported in Tables 1 and 2

Table B1. First-stage for the results reported in Table 1 of the main text.

Variables	Model A1<br>2SLS<br>Coefficients and (robust standard errors)	Model A2<br>2SLS<br>Coefficients and (robust standard errors)	Model A3<br>2SLS<br>Coefficients and (robust standard errors)	Model A4<br>2SLS<br>Coefficients and (robust standard errors)
Age	0.000	0.000	0.001	0.001
	(0.001)	(0.001)	(0.001)	(0.001)
Female	-0.018	0.012	0.016	0.017
	(0.024)	(0.034)	(0.036)	(0.036)
University education	-0.318***	-0.310***	-0.301***	-0.304***
	(0.026)	(0.037)	(0.039)	(0.039)
Married	-0.184***	-0.173***	-0.166***	-0.168***
	(0.025)	(0.036)	(0.038)	(0.038)
Unemployment	0.309***	0.240***	0.257***	0.251***
	(0.028)	(0.039)	(0.041)	(0.041)
Urban area	0.387***	0.357***	0.353***	0.330***
	(0.025)	(0.035)	(0.038)	(0.038)
Frequent unjustifiable absence of healthcare personnel		-0.052	-0.067	-0.135*
		(0.055)	(0.058)	(0.068)
Disrespectful treatment by healthcare personnel		-0.000	0.031	0.06
		(0.049)	(0.052)	(0.058)
No required drug available		0.053	0.067	0.043
		(0.042)	(0.044)	(0.052)
Healthcare facilities not clean		0.035	0.026	0.003
		(0.069)	(0.071)	(0.085)
Payments required for services which should be free of charge		-0.162***	-0.132**	-0.058
		(0.045)	(0.047)	(0.055)

Long waiting time	-0.022	-0.027	-0.017
	(0.039)	(0.042)	(0.049)
Generalized trust to other people		-0.089*	-0.017
		(0.038)	(0.042)
Trust into government		0.011	0.017
		(0.025)	(0.026)
Trust into parliament		0.022	0.009
		(0.027)	(0.028)
Trust into political parties		0.021	0.008
		(0.021)	(0.023)
Community generalized trust			-0.367***
			(0.095)
Community trust into government			-0.081
			(0.084)
Community trust into parliament			0.121
			(0.096)
Community trust into political parties			0.032
			(0.056)
Community-level frequency of unjustified absence of doctors			0.296*
			(0.138)
Community level disrespectful treatment by healthcare personnel			-0.149
			(0.137)
Community level no required drug available			0.109
			(0.101)
Community level healthcare facilities not clean			0.097
			(0.162)
Community level free service that charge for payment			-0.269*
			(0.107)
Community level into long time waiting			-0.043
			(0.094)

Household owns dwelling	-0.151***	-0.112	-0.113	-0.105
	(0.041)	(0.058)	(0.061)	(0.061)
Household has Internet access	-0.648***	-0.670***	-0.688***	-0.692***
	(0.027)	(0.038)	(0.041)	(0.041)
F-statistics of equality of all regression coefficients	103.25***	40.56***	33.24***	25.61***

Notes: Data rounded up.

Significance level: *p < 0.05; ** p < 0.01; ***p < 0.001.

Country-dummies are not shown.

Table B2. First-stage for the results reported in Table 2 of the main text.

Variables	Model A6<br>2SLS<br>Coefficients and<br>(robust standard errors)	Model A7<br>2SLS<br>Coefficients and<br>(robust standard errors)	Model A8<br>ivprobit<br>Coefficients and<br>(robust standard errors)
GDP per capita		0.000	
		(0.000)	
GDP growth rate		-0.031**	
		(0.012)	
Gini		-0.002	
		(0.005)	
Current health expenditure		0.038*	
		(0.015)	
Out-of-pocket expenditure		-0.010***	
		(0.002)	
Age	0.006***	0.001	0.001
	(0.002)	(0.001)	(0.001)
Female	-0.029	0.028	0.017
	(0.045)	(0.036)	(0.036)
University education	-0.257***	-0.297***	-0.304***
	(0.049)	(0.039)	(0.039)
Married	-0.269***	-0.196***	-0.168***
	(0.047)	(0.038)	(0.038)
Unemployment	0.226***	0.254***	0.251***
	(0.051)	(0.041)	(0.041)
Urban area	0.024	0.348***	0.330***
	(0.048)	(0.038)	(0.038)
Frequent unjustifiable absence of healthcare personnel	-0.054	-0.135*	-0.135*
	(0.085)	(0.068)	(0.068)

Disrespectful treatment by healthcare personnel	0.072	0.060	0.060
	(0.072)	(0.058)	(0.058)
No required drug available	0.143*	0.042	0.043
	(0.065)	(0.052)	(0.052)
Healthcare facilities not clean	0.028	0.008	0.003
	(0.106)	(0.085)	(0.085)
Payments required for services which should be free of charge	0.092	-0.061	-0.058
	(0.070)	(0.056)	(0.055)
Long waiting time	0.050	-0.021	-0.017
	(0.061)	(0.049)	(0.049)
Generalized trust to other people	-0.126*	-0.024	-0.017
	(0.053)	(0.043)	(0.042)
Trust into government	-0.079*	0.017	0.017
	(0.033)	(0.026)	(0.026)
Trust into parliament	-0.015	0.007	0.009
	(0.035)	(0.028)	(0.028)
Trust into political parties	-0.050	0.009	0.008
	(0.029)	(0.023)	(0.023)
Community generalized trust	-0.142	-0.308***	-0.367***
	(0.119)	(0.093)	(0.095)
Community trust into government	0.158	-0.100	-0.081
	(0.105)	(0.079)	(0.084)
Community trust into parliament	-0.211	0.021	0.121
	(0.121)	(0.094)	(0.096)
Community trust into political parties	0.029	0.066	0.032
	(0.071)	(0.056)	(0.056)
Community-level frequency of unjustified absence of doctors	-0.017	0.250	0.296*
	(0.174)	(0.137)	(0.138)
Community level disrespectful treatment by healthcare personnel	0.043	-0.132	-0.149
	(0.172)	(0.136)	(0.137)

Community level no required drug available	0.219	0.223*	0.109
	(0.126)	(0.099)	(0.101)
Community level healthcare facilities not clean	-0.681***	-0.040	0.097
	(0.204)	(0.161)	(0.162)
Community level free service that charge for payment	-0.275*	-0.171	-0.269*
	(0.134)	(0.103)	(0.107)
Community level into long time waiting	0.030	-0.016	-0.043
	(0.119)	(0.093)	(0.094)
Household owns dwelling	-0.239**	-0.114	-0.105
	(0.077)	(0.061)	(0.061)
Household has Internet access	-0.553***	-0.669***	-0.692***
	(0.051)	(0.041)	(0.041)
F-statistics of equality of all regression coefficients	20.41***	28.16***	25.61***

Notes: Data rounded up.
Significance level: *$p < 0.05$; ** $p < 0.01$; ***$p < 0.001$.
Country-dummies are not shown.
